# The utility of global longitudinal strain in the identification of prior myocardial infarction in patients with preserved left ventricular ejection fraction

**DOI:** 10.1007/s10554-017-1138-7

**Published:** 2017-04-18

**Authors:** Graham J. Fent, Pankaj Garg, James R. J. Foley, Laura E. Dobson, Tarique A. Musa, Bara Erhayiem, John P. Greenwood, Sven Plein, Peter P. Swoboda

**Affiliations:** Multidisciplinary Cardiovascular Research Centre & Division of Biomedical Imaging, Leeds Institute of Cardiovascular and Metabolic Medicine (LICAMM), University of Leeds, Leeds, LS2 9JT UK

**Keywords:** Magnetic resonance imaging, Myocardial infarction, Gadolinium, Left ventricular function

## Abstract

Prior myocardial infarction (MI) is associated with increased mortality and is prevalent in certain high risk patient groups. Electrocardiogram may be used in diagnosis, however, sensitivity is limited, thus non-invasive imaging techniques may improve diagnosis. We investigated whether global longitudinal strain (GLS) and longitudinal strain parameters are reduced in patients with prior MI but preserved left ventricular ejection fraction (LVEF). The study included 40 clinical patients with prior MI occurring >3 months previously (defined as subendocardial hyperenhancement on late Gadolinium enhancement imaging) with LVEF ≥ 55% and 40 controls matched for age and LVEF. GLS, global longitudinal strain rate (GLSR) and early diastolic longitudinal strain rate (GLSRe) were measured from cine imaging feature tracking analysis. Presence of wall motion abnormality (WMA) and minimum systolic wall thickening (SWT) were calculated from cine imaging. GLS was −17.3 ± 3.7% in prior MI versus −19.3 ± 1.9% in controls (p = 0.012). GLSR was −88.0 ± 33.7%/s in prior MI versus −103.3 ± 26.5%/s in controls (p = 0.005). GLSRe was 76.4 ± 28.4%/s in prior MI versus 95.5 ± 26.0%/s in controls (p = 0.001). GLS accurately identified prior MI [AUC 0.662 (95% CI 0.54–0.785) p = 0.012] whereas WMA [AUC 0.500 (95% CI 0.386–0.614) p = 1.0] and minimum SWT [AUC 0.609 (95% CI 0.483–0.735) p = 0.093] did not. GLS, GLSR and GLSRe are reduced in prior MI with preserved LVEF. Normal LVEF and lack of WMA cannot exclude prior MI. Prior MI should be considered when reduced GLS, GLSR or GLSRe are detected by non-invasive imaging.

## Introduction

Prior myocardial infarction (MI) is defined as either the presence of pathological Q waves on an electrocardiogram (ECG), regional loss of myocardium on cardiovascular (CV) imaging in the absence of a non-ischemic cause or pathological findings supportive of prior MI [1]. Myocardial infarction is frequently unrecognized at the time of its occurrence, accounting for 20–40% of all prior MI in high risk populations diagnosed on ECG criteria [2, 3]. Cardiovascular magnetic resonance (CMR) late gadolinium enhancement (LGE) imaging offers a more sensitive means of diagnosis than ECG and is considered the reference standard non-invasive imaging technique for the detection of prior MI [4, 5]. Studies using this approach have suggested that unrecognized MI is more common than recognized prior MI in certain populations, with a prevalence of 18% in an elderly, community-based cohort [6].

The presence of unrecognized MI detected by LGE is associated with a tenfold increase in risk of CV mortality, which appears to be incremental to conventional clinical and imaging risk factors [7]. Recognition of the condition is therefore important and secondary prevention therapy aimed at reducing long-term CV risk is recommended when prior MI is diagnosed [8].

Longitudinal strain parameters theoretically have the potential to detect prior MI. Myocardial fibers situated in the left ventricular (LV) subendocardium contribute significantly to longitudinal LV contraction in systole [9]. These fibers are susceptible to ischemia and increased wall stress [10] and hence, prior MI affecting these fibers may lead to reductions in longitudinal strain values [11]. Global longitudinal strain (GLS) is a summation of myocardial deformation in the longitudinal plane during systole [12]. It is proposed to detect subclinical LV systolic function in a number of cardiomyopathies where LV ejection fraction (LVEF) is preserved [13]. Global longitudinal strain rate (GLSR) and early diastolic longitudinal strain rate (GLSRe) represent the rate of longitudinal systolic and early diastolic deformation [12] and are comparable to tissue Doppler derived S prime and E prime measurements [14]. CMR feature tracking is an alternative method of measuring these and other myocardial strain indices and uses post-processing software to rapidly obtain measurements from steady state free precession (SSFP) cine imaging.

GLS has been demonstrated to be reduced in patients with heart failure with reduced ejection fraction (HF-REF) due to chronic ischemic heart disease [15] and in the setting of acute MI [16]. However, GLS in patients with MI but preserved LVEF has not yet been investigated.

We hypothesized that patients with prior MI detected by LGE but preserved LVEF would have impaired GLS and longitudinal strain rates compared to matched normal controls.

## Materials and methods

### Study population

This was a single centre case control study involving 40 clinical patients with prior MI occurring >3 months previously and preserved LVEF (≥55%) and 40 controls matched for age and sex with LVEF (≥55%). Prior MI patients were retrospectively recruited from consecutive patients undergoing CMR for clinical reasons and selecting those with MI and preserved LVEF. Healthy controls were prospectively recruited volunteers with no history of cardiac disease. All participants were screened for CV risk factors by completion of a health questionnaire and from medical records.

### Inclusion and exclusion criteria

Patients with significant arrhythmia (defined as uncontrolled tachycardia >100 bpm at the time of CMR study) were excluded. Prior MI patients with a history of acute MI or chest pain within the preceding 3 months were excluded on the basis that significant alterations to both myocardial scar burden and LV systolic function could have occurred in this time frame. Controls had no known history of CV disease.

### CMR acquisition

All patients underwent CMR at either 1.5 T (Philips Ingenia) or 3.0 T (Philips Achieva) and controls at 3.0 T (Philips Achieva). Images were acquired with breath holding on end-expiration prior to contrast administration and prospectively gated using a 3-lead vector ECG.

Cine images were planned from the scout images and for every patient a 2 chamber (2Ch), 4 chamber (4Ch) and an LV short axis cine stack were acquired to ensure full coverage of the left ventricle. Typical image acquisition parameters for SSFP cine acquisitions were as follows: TR 2.6 ms, TE 1.3 ms, flip angle 40°, field of view 320 × 340 mm × 100 mm, voxel size 2 × 1.62 × 10 mm, 30 cardiac phases.

LGE imaging was performed in all patients with acquisitions of a short axis LV stack, 2Ch and 4Ch obtained 10 min after administration of 0.2 mmol/kg Gadolinium DTPA contrast (Gadovist, Bayer Schering) using inversion recovery-prepared T1 weighted echo. The optimal inversion time (TI) to null normal myocardial signal ascertained by the Look Locker approach. Between 10 and 12 short axis LV, 2Ch and 4Ch images were acquired for every patient. Further imaging with altered phase-encoding direction or systolic imaging were acquired when prior MI was suspected after initial imaging.

### CMR analysis

All post processing analysis of CMR scans was performed using the same software (CVI 42, Circle Cardiovascular Imaging Calgary, Canada). LV contours were drawn manually at both end diastole and end systole on the LV short axis SSFP cine stack. LV papillary muscles were considered part of the LV cavity.

Percentage myocardial systolic wall thickening (SWT) was calculated from the end diastolic and end systolic contours as previously reported [17]. SWT was calculated for each LV segment based on the 16 segment AHA model. A cut-off value of 30% was used to define the presence of wall motion abnormality (WMA) in an individual LV segment [18]. The minimum value for each of the 16 LV segments in each patient was taken and used for comparison between prior MI patients and controls.

The presence of LGE in a subendocardial pattern suggestive of prior MI was determined independently by two physicians with over 4 years’ experience in CMR. Quantitative assessment of myocardial scar burden was performed using a threshold of 50% of the maximum intensity within the scar (full width half max method) which has been proposed as the most reproducible method for this purpose [19]. After optimization of brightness and contrast settings, manual delineation of two separate user-defined regions of interest (ROIs) were made on an LGE short axis slice where infarcted myocardium was present. One ROI was an area of hyperintense infarcted myocardium and a second ROI was drawn in remote myocardium containing no infarcted myocardium. Automated calculations for the remaining LV short axis LGE stack based on these two ROIs were then performed.

**Fig. 1:**
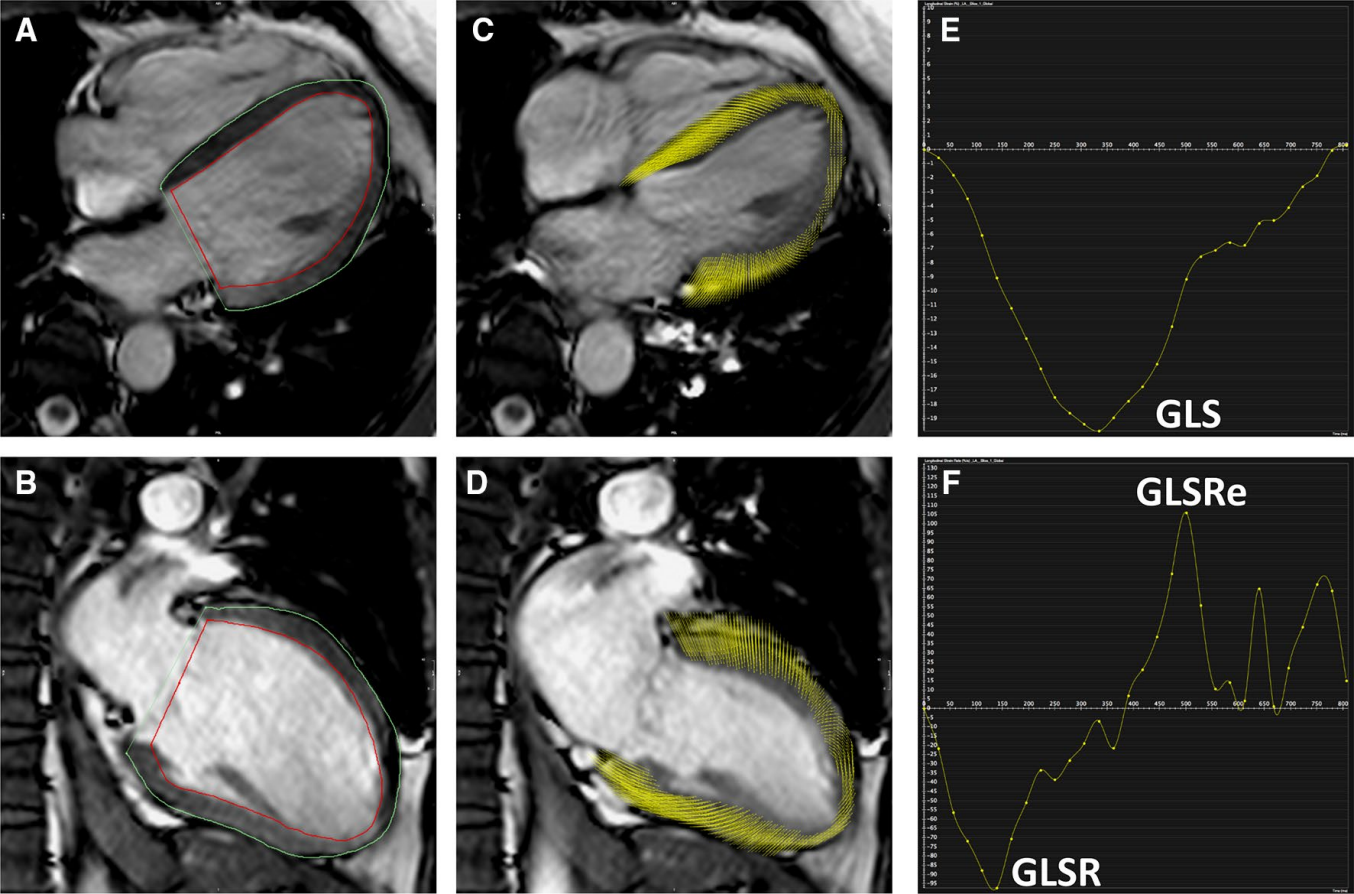
Calculation of GLS using CMR feature tracking in a healthy control. *Panel A* 4Ch cine acquisition with manually contoured epicardial and endocardial borders. *Panel B* 2Ch cine acquisition with manually contoured epicardial and endocardial border. *Panel C* feature tracking of 4Ch cine acquisition. *Panel D* feature tracking of the 2Ch cine acquisition. *Panel E* graph showing GLS [*x axis* shows time (ms) and *y axis* shows deformation (%) in longitudinal plane]. *Panel F* graph showing longitudinal strain rate [*x axis* shows time (m/s) and *y axis* shows deformation rate (%/s)]

The strain parameters GLS, GLSR and GLSRe were calculated using feature tracking software from 4Ch and 2Ch SSFP cine acquisitions (Fig. 1). Prior to analysis, brightness and contrast settings were adjusted to allow optimization of endocardial and blood pool differentiation. The epicardial and endocardial borders were traced manually. The software then tracked the voxel features of the myocardium to quantify the motion of myocardium and compute strain values [20]. This process results in generation of values of percentage deformation in the longitudinal plane throughout the cardiac cycle (providing a value for GLS) as well as deformation rate throughout the cardiac cycle (providing values for GLSR and GLSRe).

### Statistical analysis and power calculation

Normality of data was tested using a Shapiro–Wilk test. Mean values ± SD are reported. Unpaired Student *t* test and Mann–Whitney *U* test were used as appropriate to compare continuous variables. Cut-off values to identify prior MI were derived from receiver-operating characteristic (ROC) curve analysis using Youden index giving maximum sensitivity and specificity. AUCs were compared by using validated methods described by DeLong et al. [21]. Multivariable linear regression was used for variables with a statistical significance of <0.1 on univariable linear regression. Intra and interobserver variability for GLS were tested on ten randomly selected healthy controls using coefficient of variation (CoV). All tests were two-sided and p < 0.05 was considered statistically significant.

Based on the pooled standard deviation of 2.8% 31 subjects are needed in each group to detect an absolute reduction of GLS by 2% in those with chronic MI (α = 0.8, significance = 0.05).

## Results

A total of 40 prior MI and 40 healthy controls were recruited. Analysis was completed in all 40 patients in both groups, thus all were included in the final study sample. The prior MI patients were well-matched with controls for age and sex. Other variables including blood pressure, body mass index, LV mass, LV end diastolic volume (LVEDV) and LV systolic function were comparable across both groups. There were no statistically significant differences between any of these variables (Table 1).

**Table 1:** Patient characteristics in prior MI and control groups

Clinical variable	Prior MI (n = 40)	Controls (n = 40)	p value
Age	60 ± 11	57 ± 10	0.29
Females	9/40 (23%)	9/40 (23%)	–
LVEF (%)	62.3 ± 3.9	62.1 ± 3.8	0.82
LV mass (g)	107.9 ± 24.6	100.7 ± 23.8	0.18
LVEDV (ml)	159.8 ± 34.3	161.3 ± 30.2	0.84
Systolic blood pressure (mmHg)	133 ± 25.6	130.0 ± 12.3	0.61
Diastolic blood pressure (mmHg)	78 ± 19.5	73.0 ± 9.8	0.19
Body mass index (kg/m^2^)	27.9 ± 3.4	27.8 ± 3.9	0.82
Hemoglobin (g/L)	146 ± 11.5	142 ± 14.5	0.27
Creatinine (micromol/L)	77 ± 13.4	75 ± 13.6	0.52
Estimated GFR (ml/min/1.73 m^2^)	81 ± 8.5	83 ± 9.1	0.83
Hypertension	13/40 (33%)	4/40 (9%)	0.008
Hypercholesterolemia	10/40 (25%)	1/40 (3%)	0.004
Diabetes mellitus	6/40 (15%)	0/40	0.02
Smoking	18/40 (45%)	2/40 (6%)	<0.001

### Feature tracking parameters of myocardial strain

**Fig. 2:**
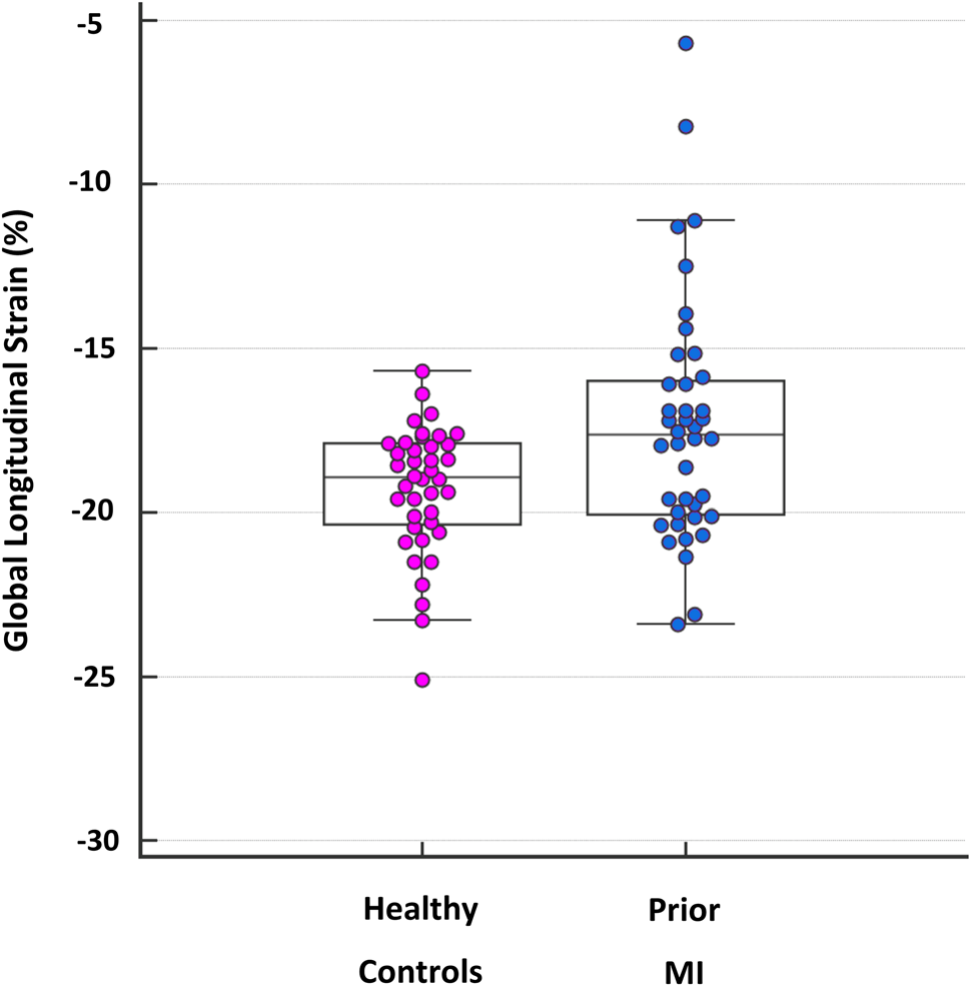
Box and whisker plot for GLS in prior MI and healthy controls. GLS values for all prior MI patients and healthy controls are shown as individual data points. For both groups, the *horizontal line in the middle of the box* demonstrates median values, the *bottom of the box* represents the 25th percentile and the *top of the box* represents the 75th percentile. The T-bar ‘whiskers’ represent the 95% confidence intervals

Feature tracking analysis was successfully performed in all prior MI patients and controls. Global longitudinal strain (Fig. 2) was significantly lower in prior MI patients than controls (−17.3 ± 3.7% versus −19.3 ± 1.9%, p = 0.012). Global longitudinal strain rate was significantly lower in prior MI patients versus controls (−88.0 ± 33.7% s^−1^ versus −103.3 ± 26.5% s^−1^ p = 0.005). Early diastolic longitudinal strain rate was also significantly lower in prior MI patients versus controls (76.4 ± 28.4% s^−1^ versus 95.5 ± 26.0% s^−1^, p = 0.001). GLS was not significantly different in prior MI patients scanned at 1.5 and 3.0 T (mean GLS at 1.5 T −18.0 ± 1.8 versus −17.2 ± 4.0 at 3.0 T, p = 0.61).

### Quantitative systolic wall thickening

Quantitative systolic wall thickening analysis was possible in all prior MI patients and controls. There was no significant difference in minimum SWT in those with prior MI compared to controls (46.0 ± 18.3% versus 42.0 ± 14.1%, p = 0.093). There was no significant difference in the proportion of subjects with WMA defined as a segment with SWT < 30% [6/40 (15%) prior MI patients and 6/40 (15%) controls].

### Receiver operator characteristic analysis

**Fig. 3:**
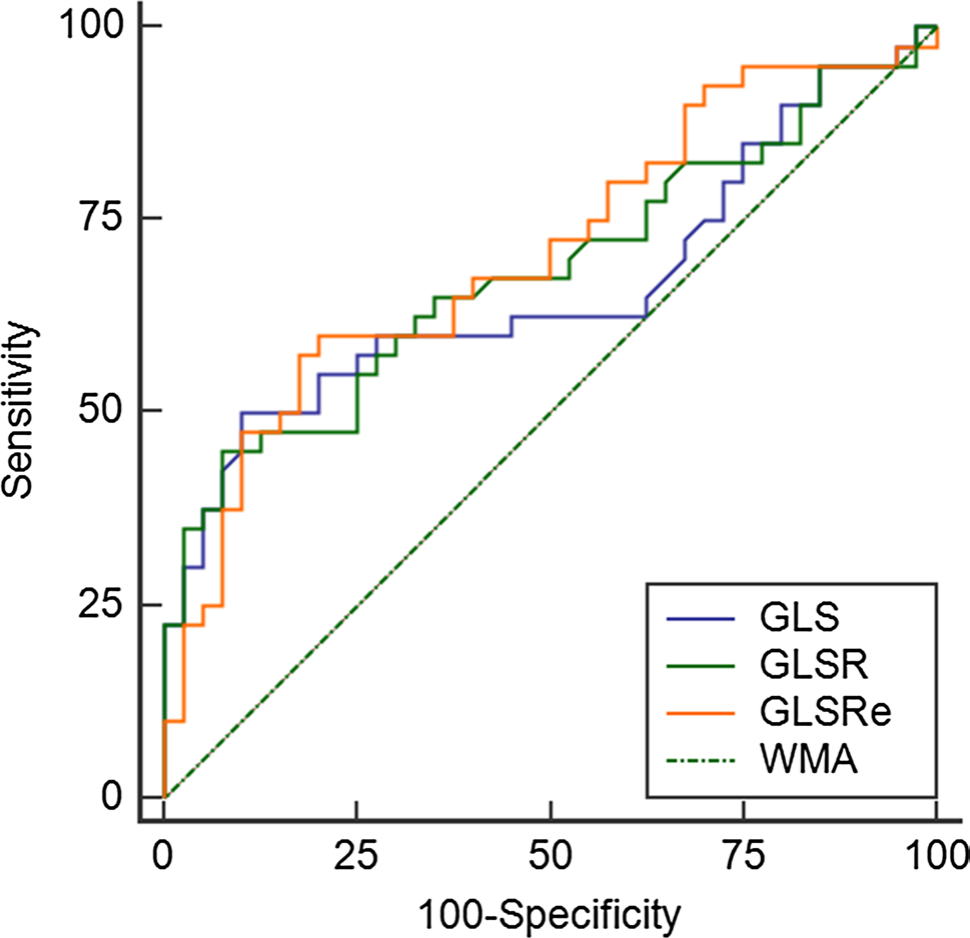
ROC curves for the accuracy of longitudinal strain parameters (GLS, GLSR and GLSRe) and presence of WMA in the prediction of prior MI

AUC for the ability of each longitudinal strain parameter to correctly identify prior MI were as follows (Fig. 3): GLS 0.662 (95% CI 0.540–0.785), p = 0.01, GLSR 0.684 (95% CI 0.566–0.802), p = 0.005 and for GLSRe 0.707 (95% CI 0.592–0.821), p = 0.001. By comparison, AUC for the ability of both presence of WMA and minimum SWT to correctly identify prior MI were lower at 0.500 (95% CI 0.386–0.614), p = 1.0 and 0.609 (95% CI 0.483–0.735), p = 0.09 respectively. Comparison of AUC values for all longitudinal strain parameters assessed showed significantly higher diagnostic accuracy for the detection of prior MI than WMA (GLS p = 0.02, GLSR p = 0.01 and GLSRe p = 0.001). Comparison of AUC values between longitudinal strain parameters and minimum SWT showed no statistical significance (GLS p = 0.59, GLSR p = 0.39 and GLSRe p = 0.29).

### Scar quantitation

Scar quantitation was successfully performed in all prior MI patients. The range of absolute scar mass was wide at between 0.7 and 21.4 g. The overall mean absolute scar mass was 5.5 ± 4.3 g, equating to a relative percentage of LV mass of 4.9 ± 3.3%.

### Sensitivity and specificity of feature tracking derived strain values

Two approaches were used in determining sensitivity and specificity for the correct identification of prior MI by the different longitudinal strain values assessed. Firstly, the cut-off value giving maximum area under the curve was determined for each variable. Secondly, the value giving maximum specificity was determined. Results are summarized in Table 2.

**Table 2:** Identification of prior MI with longitudinal strain parameters optimized for sensitivity and specificity

Variable	Sensitivity (%)	Specificity (%)	MI correctly identified	MI incorrectly identified
GLS				
Cut-off ≥18%	60	72.5	24/40	11/40
Cut-off ≥15.7%	22.5	100	9/40	0/40
GLSR				
Cut-off ≥93.5% s^−1^	65	60	26/40	16/40
Cut-off ≥66.4% s^−1^	22.5	100	9/40	0/40
GLSRe				
Cut-off <86% s^−1^	67.5	60	27/40	16/40
Cut-off <44.81% s^−1^	10	100	4/40	0/40

### Univariable and multivariable regression analysis for GLS

Variables including patient demographics, risk factors for CVD and presence of prior MI were analyzed to determine univariable predictors of GLS (Table 3). Multivariable regression analysis revealed only prior MI to be an independent predictor of change in GLS.

**Table 3:** Univariable and multivariable regression for global longitudinal strain

Univariable linear regression for global longitudinal strain			
Variable	Beta	95% CI	p value
Age	0.04	−0.03 to 0.10	0.29
Hypertension	1.26	−0.48 to 3.00	0.15
Hypercholesterolemia	1.84	−0.17 to 3.84	0.07
Diabetes	0.77	−1.96 to 3.49	0.58
Smoking (ever)	0.47	−1.16 to 2.10	0.57
Prior MI	−1.97	−3.29 to −0.65	0.004
Systolic BP	−0.01	−0.05 to 0.03	0.76
Diastolic BP	0.02	−0.03 to 0.07	0.49

Multivariable regression for global longitudinal strain			
Variable	Beta	95% CI	p value
Hypercholesterolemia	0.93	−1.11 to 2.97	0.37
Prior MI	−1.94	−3.41 to −0.47	0.01

### Observer variability

On intraobserver analysis, mean GLS values by CMR feature tracking were similar at −20.3 and −19.6% (p = 0.60) and CoV 3.9%. On interobserver analysis, mean GLS values CMR feature tracking were again similar at −20.3% and −19.6 (p = 0.62) and CoV% 4.0%.

## Discussion

We have found that in patients with preserved LVEF and prior MI there is impairment of GLS, GLSR and GLSRe. Furthermore, impairment of GLSR and GLSRe had superior diagnostic accuracy than the quantitative assessment of WMA in the detection of prior MI. These results demonstrate that prior MI may be detected when GLS is impaired when LVEF is preserved, although its ability to detect prior MI in this context is moderate.

Impairment of longitudinal measures of strain in prior MI may relate to myocardial fiber arrangement within the left ventricle. Subendocardial myofibers contribute predominantly to contraction in the longitudinal plane with subepicardial fibers providing a lesser contribution. Conversely, both circumferential and radial motion of the myocardium are generated by fibers predominantly located in the midwall [9]. Longitudinal function is particularly vulnerable to any disease process affecting the subendocardium. It is therefore possible that in limited subendocardial MI, longitudinal myocardial deformation is reduced whereas radial function, which results from contraction of LV midwall fibers, may be relatively preserved. Given that LVEF and systolic wall thickening are predominantly measures of radial contraction, this would explain the findings in this study that minimum SWT, presence of WMA and LVEF were all poor predictors of prior MI in patients with preserved LV function. Delgado et al. demonstrated that although GLS was reduced in patients with HF-REF due to chronic ischemic heart disease, the relationship between LVEF and GLS was only weakly linear (r = 0.62, p = <0.001) [15] which would again potentially support the concept of impaired GLS being largely attributable to loss of subendocardial myocardial fiber contraction.

There was no significant difference in the proportion of patients with WMA (>30% minimum SWT in any LV segments) between the prior MI and control groups. Our finding of WMA in asymptomatic controls was not unexpected and has previously been reported elsewhere in the literature [17, 22].

The importance of LVEF and regional LV systolic function as indicators of prior MI is stressed in international guidelines [1]. Our results demonstrate the potential additive value of longitudinal strain parameters and in particular GLS in looking beyond traditional means of assessing LV systolic function in the detection of prior MI.

Of the longitudinal strain parameters assessed, GLS was moderately useful in terms of specificity and sensitivity with a cut-off value of ≥18% giving a sensitivity of 60% and specificity of 72.5% and was the most useful of the 3 strain values assessed. These findings broadly correlate with those of Nucifora et al. who demonstrated a correlation between reduced GLS measured by echocardiography and the presence of significant coronary artery disease on CT coronary angiography in patients with symptoms suggestive of stable angina with normal LVEF [23]. In that study, the authors proposed a cut off value ≥−17.4% predicting significant coronary artery disease with a sensitivity of 83% and specificity of 77%. It is unclear why our own values for the sensitivity and specificity of GLS in identifying prior MI were lower, though one reason could be that ischemia has a more profound effect on GLS than infarction, possibly due to the influence of LV remodeling in the context of chronic infarction.

GLS has good inter and intra-observer variability when measured by CMR feature tracking as demonstrated in both this and another study [24]. Similar values have also been demonstrated using echocardiography [25]. The findings of this study adds to the growing evidence base supporting the use of GLS in clinical practice. It has shown potential in terms of prognostication and has been demonstrated to be superior to LVEF in predicting morbidity and mortality in patients with IHD [26]. Additionally, it shows promise as a potential screening tool for silent MI [27].

Several studies have demonstrated impairment of GLS, predominantly by speckle tracking echocardiography, in a range of disease states including diabetes [28], heart failure with preserved ejection fraction (HFPEF) [29] and aortic stenosis [30]. In these studies, impairment of longitudinal function was attributed to a direct cardiomyopathic process. However, LGE imaging was not performed in these patients and it is therefore possible that impairment of GLS may have related to unrecognized MI. Thus, future studies of the prognostic importance of GLS should include LGE imaging to exclude MI as the mechanism of impairment of longitudinal function.

Our findings support the potential utility of GLS as a screening tool for identifying prior MI in patients with preserved LV ejection fraction, although when used alone, its ability to correctly identify prior MI in this context is only moderate. Further prospective studies are needed to identify whether combining it with other imaging and/or clinical parameters lead to improvement in its specificity and sensitivity. As GLS has been shown to be impaired independently of LVEF in aortic stenosis [30], hypertrophic cardiomyopathy [31] and HFPEF [29], it must be interpreted with caution where these conditions are present or are suspected.

### Limitations of the study

The values for the strain parameters measured in this study were calculated using feature tracking post-processing software. This remains a research application and currently lacks the clinical validation to enable its adoption into routine clinical practice. Nevertheless, both GLS and GLSR can be readily measured using modern echocardiographic speckle tracking [12] which have been validated against CMR strain measurements [32].

CMR tagging has traditionally been considered the reference standard technique for calculation of strain values and was not used in this study. We have elected to use feature tracking preferentially for this study because tagging techniques suffer from both lower temporal resolution and fading of the tag overlay as the cardiac cycle progresses [24]. Furthermore agreement between feature tracking and CMR tagging is excellent [33] and can be easily performed without the need for acquisition of additional sequences.

We have not carried out invasive assessment of coronary anatomy in all patients and it is possible that undiagnosed ischaemia may have contributed to impairment of longitudinal strain parameters. However, performing coronary angiography on patients in whom it is not clinically indicated would not be ethically appropriate.

Finally, there were higher rates of CV risk factors in our prior MI population compared with healthy volunteers, thus it is unclear as to their relative contribution (if any) to the observed decrease in GLS seen in prior MI patients. Nevertherless, in our multivariable analysis, only prior MI was an independent predictor of change in GLS and CV risk factors including hypertension, smoking, hypercholesterolemia and diabetes were not.

## Conclusion

The strain parameters GLS, GLSR and GLSRe are reduced in patients with prior MI in the context of normal LVEF. A normal LVEF and lack of WMA is insufficient to exclude prior MI. Prior MI may be suspected when impaired GLS, GLSR or GLSRe are detected by non-invasive imaging.
